# Pain-preventing strategies in mammography: an observational study of simultaneously recorded pain and breast mechanics throughout the entire breast compression cycle

**DOI:** 10.1186/s12905-015-0185-2

**Published:** 2015-03-15

**Authors:** Jerry E de Groot, Mireille JM Broeders, Cornelis A Grimbergen, Gerard J den Heeten

**Affiliations:** Department of Biomedical Engineering & Physics, Academic Medical Center, Meibergdreef 9, P.O. Box 22660, 1100 DD Amsterdam, The Netherlands; Department for Health Evidence, Radboud University Medical Center, P.O. Box 9101, 6500 HB Nijmegen, The Netherlands; LRCB Dutch reference center for screening, P.O. Box 6873, 6503 GJ Nijmegen, The Netherlands; Sigmascreening B.V., Meibergdreef 45, 1105 BA Amsterdam, The Netherlands; Department of Radiology, Academic Medical Center, P.O. Box 22660, 1100 DD Amsterdam, The Netherlands

**Keywords:** Mammography, Breast, Personalized, Compression, Pain

## Abstract

**Background:**

Many women consider mammography painful. Existing studies on pain-preventing strategies only mention pain scores reported before and after breast compression. Studying the pain dynamics *during* the entire compression cycle may provide new insights for effective pain-preventing strategies.

**Methods:**

This observational study included 117 women who consented to use a custom turning knob to indicate their pain experience during standard mammographic breast compressions in the Academic Medical Center in Amsterdam, The Netherlands. The breast thickness, compression force, contact area, contact pressure and pain experience were recorded continuously. Breast volume was calculated retrospectively from the mammograms. We visualized the progression of pain in relation to breast mechanics for five groups of breast volumes and we performed multivariable regressions to identify factors that significantly predict pain experience.

**Results:**

Breast compressions consisted of a deformation phase for flattening, and a clamping phase for immobilization. The clamping phase lasted 12.8 ± 3.6 seconds (average ± standard deviation), 1.7 times longer than the 7.5 ± 2.6 seconds deformation phase. During the clamping phase, the average pain score increased from 4.75 to 5.88 (+24%) on a 0 – 10 Numerical Rating Scale (NRS), and the proportion of women who reached severe pain (NRS ≥ 7) increased from 23% to 50% (more than doubled). Moderate pain (NRS ≥ 4) was reported up to four days after the mammogram. Multivariable analysis showed that pain recollection of the previous mammogram and breast pain before the compression, are significant predictors for pain. Women with smallest breasts experienced most pain: They received highest contact pressures (force divided by contact area) and the pressure increased at the highest rate.

**Conclusion:**

We suggest further research on two pain-preventing strategies: 1) using a personalized compression protocol by applying to all breasts the same target pressure at the same, slow rate, and 2) shortening the phase during which the breast is clamped.

## Background

Millions of mammograms are made worldwide each year [1] and mammography is expected to remain the primary breast examination modality for many years. Despite continuous advances in medicine and technology, one aspect of mammography has not changed in over 50 years: the breast is still flattened onto the detector because this improves diagnostic image quality [2] and reduces dose [3]. Many women consider the so-called ‘breast compressions’ painful [4], particularly women who were conservatively treated for breast cancer [5]. Pain can deter asymptomatic women from continued breast cancer screening attendance [6] and an increasing number of women who had breast conserving surgery have little choice but to endure post-treatment follow-up mammograms [7].

Early studies mention risk factors for pain [8] to be breast tenderness, anxiety level, pain expectation and staff attitude [9-12]. Some studies also found breast density [13], breast volume [11] and menstrual status [14] to be risk factors, but other studies [11,15-17] did not support these conclusions. Several pain-preventing strategies have been proposed [18-22], but the 2008 Cochrane review [4] finds that most of these are not ready for implementation for various reasons [4,15,22-24]. Further research is continuously called for. It is remarkable though, that, to the best of our knowledge, all studies on mammography pain and pain-preventing strategies have only assessed pain levels reported directly *after* breast compression, not *during* the entire compression cycle or for some days afterwards. Studying dynamic pain experience in conjunction with breast mechanics may provide information that can be used for developing pain-preventing strategies.

One effective and widely accepted pain-preventing strategy is that the clients can request the radiographer to stop the compression when they consider the procedure too painful [9,10]. This approach could be named ‘pain-limited compression’. Another pain-prevention proposal [19] has been implemented on some mammography devices as the OpComp® feature (Siemens, Erlangen, Germany). This feature stops the compression when the breast’s ability to be flattened reaches a point where applying extra force does not result in ‘sufficient’ extra breast flattening. This breast mechanics approach could therefore be called ‘flattening-limited compression’.

We recently proposed a pain-preventing strategy [25] that aims to apply the same target *pressure* to all breasts. This would mean that the compression is stopped when the applied *force* has reached a level that is appropriate for the individual breast size: *pressure = force/contact area*. This so-called ‘pressure-standardized compression’ approach has been clinically validated and proven to avoid unnecessary pain and improve standardization without significantly affecting average glandular dose or the proportion of retakes used as a measure for diagnostic image quality [26]. It could also be called ‘personalized compression’, because the amount of compression is adjusted to the size and the mechanical properties of the individual breast.

The aim of this paper is to obtain insights on existing and potential new pain-preventing strategies in mammography by simultaneously recording pain and breast mechanics throughout the entire breast compression cycle.

## Methods

Within an observational study [7,25] approved by the ‘Medische Ethische Commissie’ (IRB) of the Academic Medical Center in Amsterdam, the Netherlands, 117 women without previous breast surgery, ages 35 – 88 years (mean 53 ± 10 standard deviation), provided written informed consent for data recording. The participants received a standard mammogram consisting of craniocaudal (CC) and mediolateral oblique (MLO) compressions. The hospital’s standard compression protocol is ‘pain-limited’, with a target force of 18 daN. This means that radiographers apply 18 daN to each breast, unless the patient requests to stop the compression at a lower force. During each compression we recorded the time-varying compressed breast thickness *Th(t)* and applied force *F(t)* from the mammography device, and the area of contact *A(t)* between the breast and the compression paddle by means of semi-automatic segmentation of video-recordings of the breast under compression [25]. The contact pressure *P(t)* is defined and calculated as the ratio of force and contact area *F(t)/A(t).* In post-processing we also assessed breast volume from the mammographic images [7].

### Dynamic pain scoring

**Figure 1:**
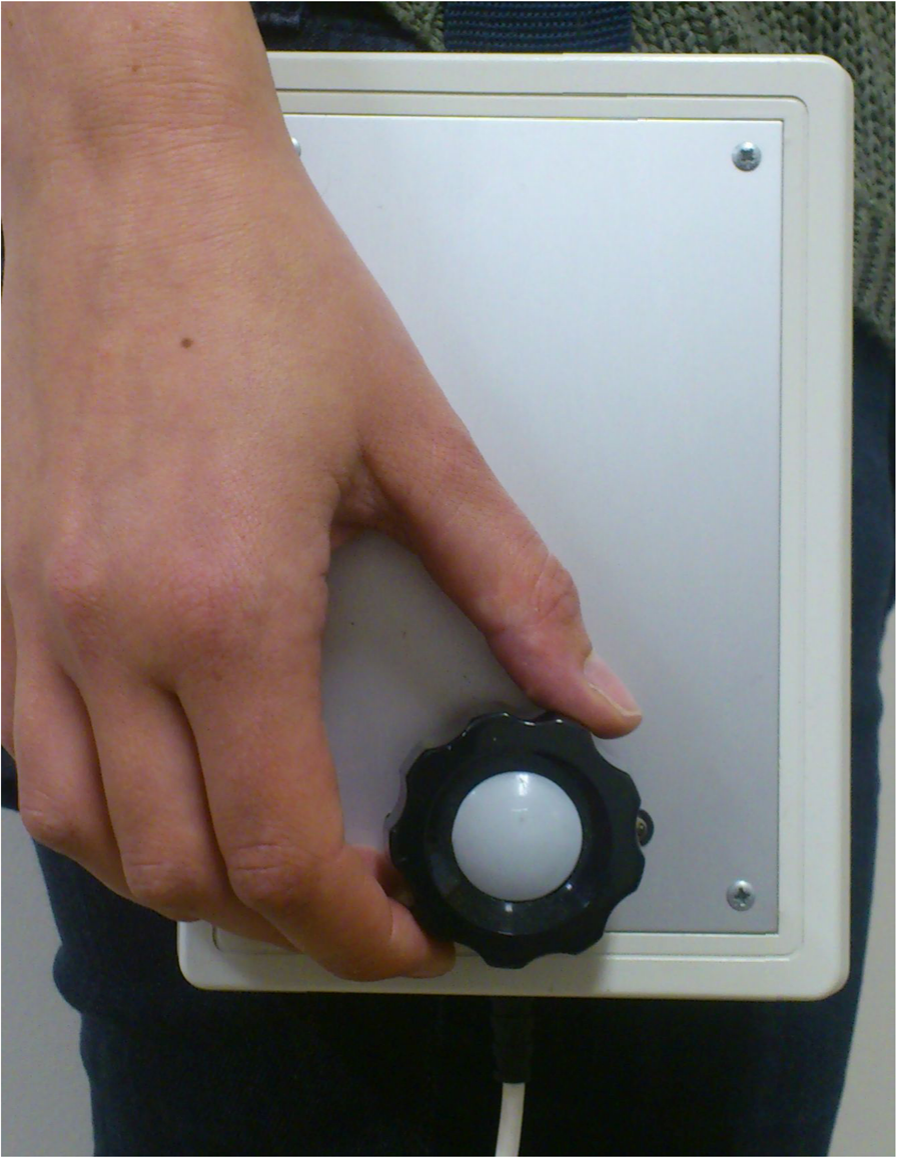
**Custom turning knob used for recording time-varying pain throughout the entire breast compression cycle.**

For recording the time-varying pain throughout the breast compressions, we developed the custom turning knob shown in Figure 1. This knob has eight discrete positions and produces an audible tone. Consecutive positions distinctly change the pitch of the tone according to the musical scale: C4 (central C), D4, E4, F4, G4, A4, B4 and C5. In the intake interviews, participants were instructed to turn the knob in the first position (C4) before each breast compression started, and then to turn it up (or down) one pitch for every ‘step’ of pain increase (or decrease) they experienced during the breast compression. In post-processing, the turning knob step signals *TK(t)* were smoothed by using linear interpolation, and normalized by dividing by the maximum step reached. This yields a *relative* pain progression curve per breast compression.

In order to compare *absolute* pain experience between individuals, participants reported their pain experience after each compression on a validated 11-point Numerical Rating Scale (*NRS*) [27]. The lowest value (0) was labeled ‘no pain’ and the highest value (10) was labeled ‘worst imaginable pain’. The turning knob’s relative pain progression curves were multiplied with the reported pain scores in order to obtain time-varying Dynamic Pain Scores *DPS(t)* that can be compared between individuals on an absolute 0 – 10 scale.

After their final compression, participants received a diary for reporting any remaining pain up to five days afterwards. During the intake interview shortly before receiving their mammogram, they were also asked about menstrual status and any baseline pain present before the compression. Returning patients were also asked to recollect the *NRS* pain score of their previous mammogram.

### Analyses

For a total of 324 breast compressions, we simultaneously recorded mechanical parameters and dynamic pain scores. To study the personalized compression approach of applying the same target pressure to all breasts [25], we stratified the sample into five equally sized groups based on breast volume: 65 very small breasts (0.20 – 0.52 dm^3^), 65 small (0.53 – 0.78 dm^3^), 65 medium (0.79 – 1.02 dm^3^), 64 large (1.03 – 1.34 dm^3^) and 65 very large breasts (1.35 – 3.00 dm^3^).

In mammographic breast compression we distinguished two phases: a deformation phase to make the breast flatter, and a clamping phase to immobilize the breast until x-ray exposure has finished. The start of the deformation phase (*t = 0* ) could not be clearly defined because the radiographer is positioning the breast. As part of the data processing, one researcher (JdG) manually selected the start of all compressions based on two criteria: (1) the applied force was between 2 and 5 daN, and (2) an initial breast-paddle contact area was present but not larger than 50% of the final contact area. The end of the deformation phase equals the start of the clamping phase and is defined as the moment at which the maximum applied force is reached for the first time. The clamping phase ends when the paddle automatically begins to move upward after the x-ray exposure has finished. Since breast compressions had different durations, we compared the recorded data on a relative time-scale, i.e. as fractional progression of the deformation and clamping phases.

Results were visualized as trendlines for the mean with 95% confidence interval for breast thickness, contact area, compression force, contact pressure and dynamic pain scores, as well as corresponding boxplots at the end of the clamping phase to give an impression of the data spread. We also presented boxplots for the recollected pain scores of the previous mammogram and for the pain scores reported up to five days after the mammogram. Since average pain scores were very similar in the CC direction (5.88; n = 161) and MLO direction (5.87, n = 163), these were visualized together. In the figure panels for contact pressure, a gray horizontal bar is included as reference for the range of normal blood pressures.

Multivariable regression analyses were performed to assess which of the predictors; recollected pain score of the previous mammogram, baseline pain score, menstrual status, compressed breast thickness, compression force, compression pressure, contact area, breast volume, deformation phase duration and clamping phase duration; significantly (p-values < 0.05) predict the pain scores reported after each compression. Model selection (in- and exclusion of predictors) was performed automatically by minimizing the Akaike Information Criterion (AIC) using the bi-directional stepwise algorithm ‘step’ (R version 3.1, R Foundation for Statistical Computing, Vienna, Austria).

## Results

### Pain experience

Stepwise multivariable regression analyses resulted in an AIC-minimized model in which only the recollected pain score of the previous mammogram was a highly significant (p < .001) predictor for the reported pain scores (Model 1 in Table 1). It is noted however that the recollected pain scores had been asked during the intake interview only shortly (<30 minutes) before the pain scores for the actual breast compressions were asked. The Pearson’s correlation coefficient was *r* = 0.72, p < .001.

**Table 1:** **Multivariable models with general shape: Pain after compression = Σ Effect × Predictor value**

	**Model 1 (n = 281)**		**Model 2 (n = 324)**	
**Predictor**	**Effect**	**Significance**	**Effect**	**Significance**
Recollected pain score [NRS]	0.763	p < .001	*excluded*	
Baseline pain score [NRS]	0.108	p = .06	0.263	p < .002
Maximum reached force [daN]			−0.204	p < .005
Duration of clamp phase [sec]	−0.046	p = .10	−0.075	p < .05

Model selection was performed by stepwise minimization of the Akaike Information Criterion. Model 1 includes the recollected pain scores of the previous mammogram (available for n = 281 cases). In model 2 (all cases: n = 324), the recollected pain scores were excluded.

We therefore also performed stepwise multivariable regression analyses excluding recollected pain score as a predictor. This resulted in Model 2 in Table 1. The baseline pain score for any pain present before the breast compression was a positive and highly significant predictor. The maximum reached compression force and the duration of the clamping phase were significant predictors with a negative effect on compression pain. The first is in agreement with the pain-limited compression protocol used: when the patient indicated that she considered the procedure painful, the radiographer applied less force. The second cannot be explained by the protocol itself, but possibly the radiographers tried to work faster knowing that the patient is in pain.

Table 2 shows pain experiences at different moments, between first-time and returning patients and between the five groups of women with different breast sizes. We note that first-time and returning participants have very similar experiences. None of the differences reached statistical significance. For the stratification by breast volume there was no consistent trend: women with smallest breasts reported highest pain, women with medium sized breasts reported least pain and women with large and very large breasts scored in between. The latter group reported most pain in the three days after the mammogram. None of the differences between adjacent volume groups reached statistical significance.

**Table 2:** **Pain outcomes (averages and proportions) at different moments and compared between first-time and returning participants and between five breast volume stratifications**

	**Attendance**		**Total**	**Stratification by breast volume**				
	**First time**	**Returning**		**Very small**	**Small**	**Medium**	**Large**	**Very large**
**Average pain scores**	n = 43	n = 281	n = 324	n = 65	n = 65	n = 65	n = 64	n = 65
Recollected from previous mammogram	-	6.19	6.19	7.04	6.72	5.51	5.07	6.28
At end of deformation phase	4.40	4.80	4.75	5.45	5.03	4.19	4.39	4.65
At end of clamping phase	5.70	5.90	5.88	6.70	6.15	5.03	5.75	5.75
**Proportions severe pain** (*NRS ≥ 7* )								
At end of deformation phase	.16	.24	.23	.32	.28	.15	.19	.22
At end of clamping phase	.51	.49	.50	.58	.55	.34	.52	.49
**Proportions moderate pain** (*4 ≤ NRS < 7* )								
1 day after mammogram	.05	.15	.11	.13	.13	.06	.17	.18
2 days after mammogram	.05	.05	.04	.03	.08	.02	.03	.10
3 days after mammogram	0	.04	.03	.03	.03	0	0	.10
4 days after mammogram	0	.01	.01	.03	0	0	0	0
5 days after mammogram	0	0	0	0	0	0	0	0

As for the progression in time, we note that during the clamping phase, the average dynamic pain score for the entire sample increased from 4.75 to 5.88 (+24%), and the proportion of women experiencing severe pain (*DPS ≥ 7* ) increased from 23% (n = 75/324) to 50% (n = 161/324). Thus, from the moment when the radiographer stops the compression, dynamic pain scores still increase more than one full NRS-point and the number of patients who experience severe pain more than doubles.

For the five days after the mammograms, moderate pain scores (*4 ≤ NRS < 7* ) were reported up to four days. For the entire sample, there is a low but significant correlation between experiencing severe pain during the compression and experiencing moderate pain on the day after the mammogram: Pearson’s *r* = 0.24, p < .001.

### Compression mechanical parameters

**Figure 2:**
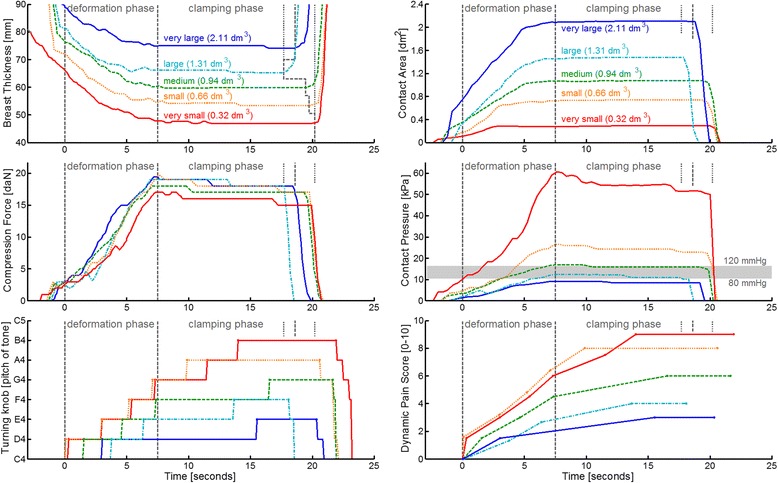
**Selected examples of raw data recordings for five breast volumes: Actual-time progression of the recorded variables and the dynamic pain score.** The gray vertical dashed lines indicate the deformation and clamping phase. For reference: the gray horizontal bar in the middle-right panel indicates the range of normal blood pressures.

**Figure 3:**
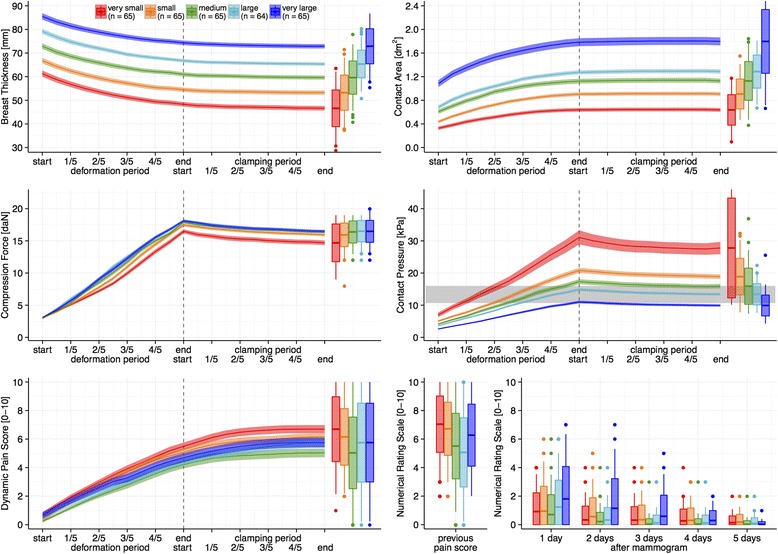
**Mean ± 95% confidence interval for all 324 compressions stratified by breast volume into five equally sized groups: Progression of the recorded variables and the dynamic pain scores on a relative time scale.** Boxplots represent the distributions of values at the end of compression as well as (bottom right) for recollected pain scores of the previous mammogram and for pain scores five days after the mammogram. Boxes are mean ± one standard deviation and whiskers extend until the furthest outlier with a maximum of one standard deviation. For reference: the gray horizontal bar in the middle-right panel indicates the range of normal blood pressures.

In Figures 2 and 3, we observe that the breast volumes, ranging from very small to very large, have a corresponding order in compressed breast thickness: from thinnest to thickest; and in contact area: from smallest to largest. We found significant correlations between breast dimensions: Pearson’s *r* values with breast volume are 0.77 (thickness) and 0.90 (contact area), both p < .001.

In the middle-left panel of Figure 3 we note that the 18 daN target force was approached for all breast sizes, except for women with very small breasts. More women in this latter group reported high pain scores and had requested the radiographers to stop the compression at a lower force. In the middle-right panel we note that the corresponding pressures differ greatly, ranging from 5 to 115 kPa. The pressure values exhibit a clear trend with respect to breast volume: from an average of 10 kPa (75 mmHg) for very large breasts to an average of 30 kPa (225 mmHg) for very small breasts.

**Figure 4:**
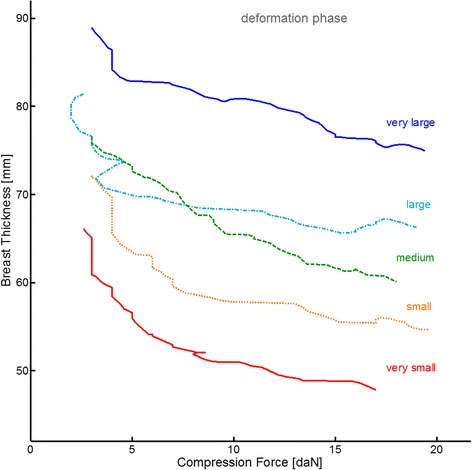
**Breast deformation curves for five selected example compressions.** The curves are obtained by plotting the *Th(t)-*values from the deformation phase as function of the corresponding and simultaneously recorded *F(t)*-values.

The deformation phase lasted on average 7.5 ± 2.6 seconds standard deviation. During this phase, the breast is flattened by applying an increasing amount of force. In Figures 2 and 3 we observe that the thickness curves gradually become more horizontal, which indicates that the gain in breast flattening becomes less with increasing force. To further illustrate this characteristic trend, the time-varying thickness and force data can also be rearranged to construct the breast specific deformation curves *Th(F)* shown in Figure 4. The slopes of these curves indicate the amount of reduction in breast thickness per additional unit of force. The general trend starts steeply downward (easily flattened) and gradually become less steep (more difficult to flatten).

The clamping phase lasted on average 12.8 ± 3.6 seconds standard deviation, which is 1.7 times longer than the deformation phase. During the clamping phase, the position of the paddle is fixed so that the breast is held still between the paddle and the detector. Despite the absence of paddle movement, we observe in Figures 2 and 3 that the thickness continues to decrease, on average 1.51 mm (2.5%). The contact area increases on average 0.07 dm^2^ (0.6%), and the force and pressure decrease substantially during the clamping phase, respectively 1.6 daN (9.3%) and 1.7 kPa (9.1%). This particular effect is known as creep motion, which is characteristic for viscoelastic soft tissues under compression [28,29]. From Figures 2 and 3 it can be observed that the decrease in force and pressure is steepest (quickest) directly after having reached the maximum force, and then gradually becomes less steep (slower). The quick component of the creep motion is attributed to effusion of blood from the breast, while the slow creep component is attributed to stretching of the collagen matrix [29].

## Discussion

In this study we simultaneously examined pain experience and breast compression mechanics in mammography in two ways: 1) as function of breast size (stratification by volume), and 2) as progression in time (during and after the compression). From our results we can propose two possible approaches for pain-preventing strategies:

### Personalized compression by using a target pressure

In the daily practice of this observational study, a compression protocol with a target force has been used. Women received similar maximum forces, but because of differences in individual breast size, the applied pressures varied greatly. Women with smallest breasts received highest pressures and also reported most pain.

We recently proposed a pain-preventing strategy [7,25] that aims to personalize compression by using a target pressure. From the physics equation—Pressure [kPa] = Force [daN] / Contact area [dm^2^]—we note that applying the same pressure to each breast means that the amount of force has to be made proportional to the individual breast contact area. In other words: the compression is personalized because small breasts will reach the target pressure at a lower force than large breasts do. This can also be checked in the middle-right panel of Figure 3 by noting that the level of normal diastolic blood pressure, i.e. the bottom of the horizontal gray bar, is reached after approximately 1/5 of the deformation phase for very small breasts, after 2/5 for small breast, 1/2 for medium, 3/5 for large and at the end for very large breasts. As a result, the rate at which pressure is applied during the compression is much higher for very small breasts (average 4.3 ± 3.0 kPa/sec standard deviation) compared to very large breasts (1.5 ± 0.7 kPa/sec). Applying pressure at a higher rate might cause more pain. We would suggest personalizing breast compression by using a standardized target pressure and a standardized rate of applying pressure.

### Shorter clamping phase

During the clamping phase, pain scores continued to increase by 24% and the number of women who reach the level of severe pain more than doubled. This phase lasted 12.8 seconds on average, during which the radiographers had to take several steps to get behind the protective glass console to press the exposure button. At that moment, the x-ray tube spins up the anode target to prevent overheating, performs a pre-shot to determine exposure settings, adjusts the target and filter if necessary, and eventually performs the x-ray exposure. There seem to be ample possibilities to shorten this phase. Mammography devices could be ready for exposure much earlier, and, with appropriate radiation protection, radiographers could stand closer to the client and use a remote control to trigger the exposure. From our data we calculate that, if the clamping phase could be shortened to one third, that is, 4.3 seconds instead of 12.8, the average pain scores would become 5.48 instead of 5.88, and the proportion of women who experience severe pain would become 37% (n = 212/324) instead of 50%. There may be additional psychological pain relief effects when the radiographer stays closer to the client and when the ‘worst part’ of the compression is over very soon after the preparatory deformation phase. Further research is needed to determine whether creep motion leads to more image blurring when a shorter clamping phase is used.

### Considerations regarding existing pain-preventing strategies

‘Pain-limited compression’ is a widely implemented pain preventing strategy in which the radiographer stops the compression when the client requests so because the procedure has become too painful. Our dynamic pain measurements show that, between the moment of stopping the compression and releasing the breast, the pain scores on average still increase by 24%. So, even though stopping the compression would prevent going beyond the client’s pain tolerance *at that specific moment*, during the 12.8 seconds of an average clamping phase it may well be exceeded. A shorter clamping phase would provide a solution.

The ‘flattening-limited compression’ strategy is based on the fact that the amount of breast flattening per unit of applied force gradually decreases: at some point, applying more force will not make the breast *sufficiently* flatter. The threshold value based on which this approach determines to stop the compression is defined as a predetermined value of the slope of the breast specific deformation curve *Th(F)*. However, we notice in Figure 4 that these slopes are typically not smooth and not continuously decreasing. This is because the radiographers sometimes have to hold the paddle still or move it upwards to properly position the breast, and because they also have to pull the breast onto the detector to maximize the amount of projected tissue. With these routine disturbances in the thickness and force signals, the slope of the *Th(F)* curve may not be a reliable representation of the breast specific deformation. It is therefore questionable whether flattening-limited compressions can be performed in a reproducible way. Pressure-standardized compression does not depend on the *slope* of the measured signals but on the measured pressure-signal itself. Using a target pressure would therefore be more robust than the flattening-limited approach.

### Comments on dynamic pain measurement

We recorded dynamic pain experience with a custom-built turning knob that produces an audible tone as feedback to the woman. There is a validated continuous pain score meter available, based on the Visual Analogue Scale [30], but we considered this method unsuitable because of the specific positions in which women are positioned during their mammographic examination. A handheld grip strength sensor may be a more intuitive method for measuring pain.

We also performed an objective pain measurement by analyzing the electro-dermal activity (EDA) between the index and middle finger of the hand that was not used for the turning knob. A similar method is used in the polygraph and for assessing time-varying pain experience in patients who are unable to verbally express themselves [31]. Unfortunately, the EDA-signals we measured were noisy, and counting the number of signal fluctuations per minute [32] did not give consistent results (data not shown). We believe that limited time for establishing a baseline signal, together with movement of the client, may have disturbed our EDA-measurement.

To the best of our knowledge, this is the first study in which remaining pain was measured for five days after the mammogram. The observation that there are some women who experience moderate pain (*4 ≤ NRS < 7* ) up to four days afterwards is at least remarkable.

### Limitations

Our multivariable analyses showed that the recollected pain score from the previous mammogram was the only significant ‘predictor’ for the compression pain score, although it is more accurate to consider it a ‘correlation’ because the causality is unclear and complex. There are also objective patient characteristics (e.g. very small or sensitive breasts), subjective patient characteristics (e.g. psychology or personal attitude), or anticipation related to a previous negative experience, which could all explain why certain women may have a predisposition towards always regarding mammography painful. It is noted that the highly significant correlation (*r* = 0.72, p < .001) found in our data is much higher than the *r* values from 0.39 to 0.50 reported in literature [33]. An explanation is that the latter are correlations between pain experiences that were actually scored at 2-year interval mammograms, whereas in our case, the previous pain scores were ‘recollections’ asked only shortly (<30 minutes) before the actual breast compressions. This, and also the fact that participants had to cooperate both during the procedure and in the following days, may have resulted in a psychological attitude focused on pain, which may have increased their pain sensitivity.

In our study we translated the individual pain scores into grouped pain progression curves. This was possible because of the extraordinary nature of our study where data were available during the compression cycle. However these scores are individual and subjective and as such not suitable for standardization per se.

In our analyses of pain we have not included any psychological or emotional factors related to the patient, the radiographer or their interaction. The importance of these factors has been emphasized in literature [10,34] and we acknowledge that the ability, deftness, kindness and attitude of the radiographer may be the first and most important tool to prevent and reduce pain.

### Recommendations

Combining the results of this study we propose further research on two pain-preventing strategies: 1a) applying the same maximum pressure to all breasts (i.e. allowing lower pressure on the woman’s request) - a contact pressure of 10 kPa (75 mm Hg) is expected to correspond with normal diastolic blood pressure inside the breast; 1b) applying that pressure at a constant rate, which effectively means that the deformation phase has the same duration for everyone; 1c) applying that pressure at a rate that is slow enough to allow blood effusion and collagen matrix stretching to happen gradually so that creep motion during the clamping phase is minimized; 2) optimizing the workflow and mammography device to achieve a substantial shortening of the clamping phase.

We must also remember that the recollected pain score of the previous mammogram is significantly correlated to the current pain score, and likely also a good predictor for future pain scores. Avoiding unnecessary pain by the mentioned strategies at the first mammogram will be important for reducing pain in the long run.

## Conclusion

To the best of our knowledge, this is the first study in which pain score is measured continuously throughout mammographic breast compressions, alongside with breast thickness, compression force contact area and contact pressure. Results suggest two potential pain-preventing strategies: 1) using a personalized compression protocol by applying to all breasts the same target pressure at the same, slow rate, and 2) shortening the phase during which the breast is clamped.
